# Estimation of the Maternal Investment of Sea Turtles by Automatic Identification of Nesting Behavior and Number of Eggs Laid from a Tri-Axial Accelerometer

**DOI:** 10.3390/ani12040520

**Published:** 2022-02-20

**Authors:** Lorène Jeantet, Vadym Hadetskyi, Vincent Vigon, François Korysko, Nicolas Paranthoen, Damien Chevallier

**Affiliations:** 1 Institut Pluridisciplinaire Hubert Curien, CNRS–Unistra, 67087 Strasbourg, France; damien.chevallier@cnrs.fr; 2 UFR Math-Info, Université de Strasbourg, 7 rue Descartes, CEDEX, 67081 Strasbourg, France; vadym.hadetskyi@gmail.com (V.H.); vigon@math.unistra.fr (V.V.); 3 Office Français de la Biodiversité, Direction des Outre-mer, Délégation Guyane, 44 rue Pasteur, BP 10808, 97338 Cayenne, France; francois.korysko@onf.fr (F.K.); nicolas.paranthoen@onf.fr (N.P.); 4 BOREA Research Unit, National Museum of Natural History (MNHN), UMR CNRS 7208, Sorbonne Université, French Institute for Research and Development (IRD 207), University of Caen Normandie, University of Antilles, CEDEX 05, 75231 Paris, France

**Keywords:** accelerometer, bio-logging, V-net, *Chelonia mydas*, behavioral classification, ecology, deep learning, conservation, convolutional neural network

## Abstract

**Simple Summary:**

During the reproduction period, female sea turtles come several times onto the beaches to lay their eggs. Monitoring of the nesting populations is therefore important to estimate the state of a population and its future. However, measuring the clutch size and frequency of sea turtles is tedious work that requires rigorous monitoring of the nesting site throughout the breeding season. In order to support the fieldwork, we propose an automatic method to remotely record the behavior on land of the sea turtles from animal-attached sensors; an accelerometer. The proposed method estimates, with an accuracy of 95%, the behaviors on land of sea turtles and the number of eggs laid. This automatic method should therefore help researchers monitor nesting sea turtle populations and contribute to improving global knowledge on the demographic status of these threatened species.

**Abstract:**

Monitoring reproductive outputs of sea turtles is difficult, as it requires a large number of observers patrolling extended beaches every night throughout the breeding season with the risk of missing nesting individuals. We introduce the first automatic method to remotely record the reproductive outputs of green turtles (*Chelonia mydas*) using accelerometers. First, we trained a fully convolutional neural network, the V-net, to automatically identify the six behaviors shown during nesting. With an accuracy of 0.95, the V-net succeeded in detecting the Egg laying process with a precision of 0.97. Then, we estimated the number of laid eggs from the predicted Egg laying sequence and obtained the outputs with a mean relative error of 7% compared to the observed numbers in the field. Based on deployment of non-invasive and miniature loggers, the proposed method should help researchers monitor nesting sea turtle populations. Furthermore, its use can be coupled with the deployment of accelerometers at sea during the intra-nesting period, from which behaviors can also be estimated. The knowledge of the behavior of sea turtle on land and at sea during the entire reproduction period is essential to improve our knowledge of this threatened species.

## 1. Introduction

Estimation of parental investment in sea turtles relies primarily on the measurement of reproductive outcomes of females. Without parental care, female sea turtles favor energy investment in pre-ovipositional allocations and lay several nests of 50 to 130 eggs per breeding season depending on the species [1]. Inter and intra-individual variations in the number of clutches and of eggs laid during a breeding season have been observed within populations suggesting variation in energy invested in the offspring [2,3,4]. Therefore, measuring clutch size (i.e., number of eggs laid) and clutch frequency (i.e., number of clutches per breeding individual) can be used as indicator of maternal investment in sea turtles. However, both of these parameters are difficult to obtain by long-term population monitoring.

Measuring the clutch size and frequency of sea turtles is tedious work that requires rigorous monitoring of the nesting sites throughout the breeding season. The most common method is based on capture–mark–recapture design: patrols of at least eight hours are carried out every night to survey the nesting sites and identify every female that comes ashore, using a Personal Integrated Transponder(PIT) tag or an unique numbered flipper tag [5,6,7,8]. However, this method requires a consequent number of observers performing long continuous trips to cover the entire beach and ensure that no individuals are missed, and thus is an important logistic with expensive costs. Moreover, since it is difficult not to miss any sea turtle, the observed number of clutches deposited by sea turtles is generally lower than the real number [5,9,10]. The number of eggs laid is even more complicated to obtain, as it requires observers to stay with one turtle for almost the entire nesting process counting the deposited eggs [11]. Finally, the capture–mark–recapture monitoring method is impractical for a large population or extensive area. Therefore, there is a crucial need to develop an efficient method to remotely record reproductive outcomes of sea turtles in order to support the intense monitoring effort of field observation.

Few studies have explored the use of new technologies to record reproductive outcomes of nesting sea turtle populations. For example, Blanco et al. [12] used ultrasonography of females’ ovaries to visualize their reproductive stage. Ultrasound scans allowed them to identify the remaining number of clutches of every scanned female and thus obtain a more accurate clutch-frequency estimation. However, it was not possible to estimate the number of eggs laid from this method and night patrols were still required [12]. In addition, ultrasonography requires direct and repeated interference with the turtles, which may disturb the animals and affect the nesting process while making it difficult to apply over large geographic areas. Another way to estimate clutch frequency of sea turtles relies on deployment of animal-attached tags throughout the breeding season [8,13,14]. Therefore, Weber et al. [8] tested a combination of Very High Frequency (VHF) radio-telemetry and Argos-linked Fastloc Global Positioning System (GPS) tags. Although VHF transmitters are low cost, they still required direct observations of the females and were ineffective at distance > 1 km. On the other hand, GPS tags allowed remote monitoring and were accurate enough to locate individuals on the beach. However, the location appearing on the beach does not guarantee successful nesting, given the possible abortion of nesting without laying eggs and the large number of U-turns (also known as false crawls) undertaken by sea turtles, especially green turtles (Chevallier, personal observation) [10,15]. In addition, the high cost of Argos-linked Fastloc GPS tags limits their use and the number of equipped females [8].

Accelerometer is a low-cost miniature sensor that can provide high-frequency information about the body movements and postures of animals to which it is attached. It measures static and dynamic acceleration and enables researchers to remotely deduce behaviors for animals that are difficult to observe. Over the past few years, there has been an explosion of its use on both terrestrial and marine species [16], for which direct observations are impracticable. Therefore, a few studies monitored the underwater behavior of sea turtles from accelerometers [17,18,19,20], but their interpretation remains difficult without rigorous validation and limits their use on these species [21,22]. Only one study refers to the identification of the nesting behavior of sea turtles from accelerometer [23], while visual validation of acceleration signals is easier to achieve on land than at sea. Such method could be complementary to lighter population monitoring by indicating when and how many times an equipped sea turtle would have come to nest on the beach throughout the breeding period.

The aim of this experimental study is to evaluate the extent to which the accelerometer can remotely measure reproductive output of sea turtles. First, we deployed accelerometers on 14 nesting green turtles and visually monitored their behavior simultaneously. Next, we used this dataset to validate the identification of their nesting behavior from acceleration signals and train a powerful supervised learning algorithm to perform it automatically. For this purpose, we tested a fully convolutional neural network that had already proven effective in automatically identifying the underwater behavior of green turtles [24]. Finally, we tested whether it is possible to estimate the clutch size from the acceleration signal.

## 2. Materials and Methods

### 2.1. Data Collection

The field work was carried out in April 2019 at Awala-Yalimapo beach (5.7° N, –53.9° W), French Guiana, South America. We deployed CATS (Customized Animal Tracking Solutions, Oberstdorf, Germany) devices including tri-axial accelerometers on 14 free-ranging adult female green turtles during the nesting process. The acceleration was recorded at a frequency of 20 Hz for the three body axes of the sea turtle (AccX: back-to-front axis, AccY: left-to-right axis and AccZ: bottom-to-top axis). The devices were fixed to the turtle’s carapace by four suction-cups allowing us to rapidly operate with minimum disturbance. It took less than a minute to attach the device. In most case, we spotted the turtle going up the beach and waited for its first sand-sweeping to start (see Section 2.2 for further description of sand-sweeping and other nesting behaviors). If the turtle did not seem stressed or was not surrounded by group of humans (adding a source of stress), we quickly set the device during this step on the front of the carapace. Otherwise, we waited until the turtle began digging or even laying their eggs. For the 14 turtles (Table 1), and during the laying process, we checked, using a manual reader (GR250, TROVAN^®^, Douglas, Isle of Man, British Isles), the presence of a Passive Integrated Transponder (PIT) or injected a new one into the right triceps of the unknown turtles. We measured their Curved Carapace Length (CCL) and Curved Carapace Width (CCW) as described in Bonola et al. [25]. In parallel, the behaviors were visually monitored by an assigned person who recorded the corresponding executed time on a voice recorder. For eight nesting green turtles, for whom a good visualization of the eggs allowed it, an observer counted the exact number of eggs laid per contraction and dictated it to a second person who recorded it with the exact observation time in a voice recorder. The position of a few of the turtles did not allow us to record the number of eggs without disrupting them. So for them, we did not count the laid eggs.

### 2.2. Labelling of Nesting Behaviors

**Figure 1:**
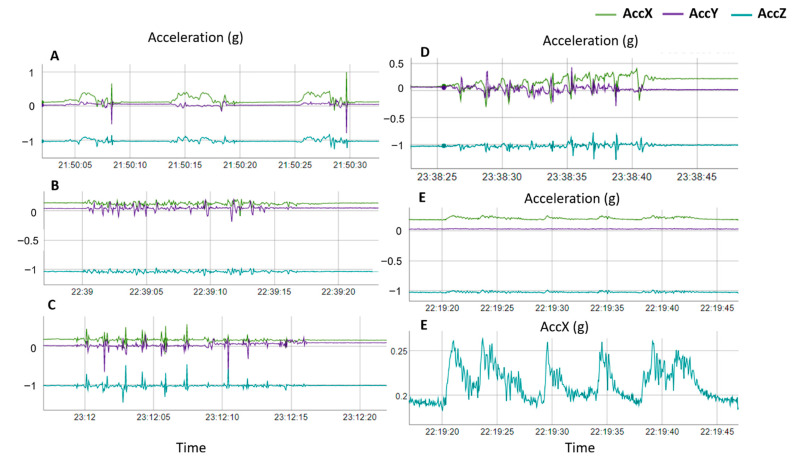
Acceleration signals corresponding to the five behavioral categories of nesting green turtle: Digging (**A**); Covering (**B**); Sand-sweeping (**C**); Walking (**D**); and Egg laying (**E**). We also represent the X-axis of the acceleration of Egg Laying. AccX corresponds to acceleration of the back -to-front body axis, AccY to the left-to-right axis and AccZ to the bottom-to-top axis.

The nesting behaviors of the sea turtle are similar between the species and the different phases and action patterns were precisely described in several ethograms [26,27,28,29]. In this study, we focused on the action patterns that resulted in different acceleration signals and thus identified five behaviors: Sand-sweeping, Digging, Egg laying, Covering, and Walking. Based on the definitions and the characteristics given by Lindborg et al. [28], Sand-sweeping corresponds to the “Body Pitting” and “Camouflaging” phases described in their article since both behaviors encompass the same movements, Digging includes the “Transition period”, and Walking represents all the forward movements, as described in the “Ascent” phase in their article. We synchronized the observation time of the behaviors with the acceleration data and visualized them using a rblt package ([30], Figure 1). Throughout the nesting process, green turtles expressed numerous latency periods inter-cutting the behaviors with easily noticeable flat acceleration signals. Therefore, we labelled them from the visualisation of the acceleration signal with an additional behavior: Motionless (Figure 1).

### 2.3. Automatic Behavioral Identification through Deep Learning

In order to automatically identify the six nesting behaviors from the accelerometer, we trained a fully convolutional neural network: a V-net. The latter was originally developed by Milletari et al. [31] for biomedical 3D image segmentation and an adapted version for the behavioral identification from time series data was tested on underwater free-ranging green turtles and revealed to be efficient [24]. A precise description of the algorithm as well as the processing steps are detailed in Jeantet et al. [24]. Before training the algorithm, we reduced the noise of the acceleration signals on the three axes (AccX, AccY, and AccZ) with a low pass band butterworth filter at 2 Hz and computed the Dynamic Body Acceleration (DBA) from the smoothed signals as described in Jeantet et al. [22]. We randomly split the 14 green turtles into three distinct groups to perform the training/validation/testing datasets. Firstly, when fed with the four previously described descriptors (the smoothed AccX, AccY, AccZ and DBA), the V-net is trained and tuned on eight randomly chosen green turtles and validated on three other individuals. We balanced the behavioral labels in the data batch through a biased random draw of the windows. In particular, we chose a lower probability of randomly drawing Motionless, which is the most frequent behavior. The training and tuning process allowed us to set up the hyper-parameters of the algorithms (depth = 12, window-size = 40, batch = 200 and learning rate = 0.01) and revealed some important confusion between Egg laying and Motionless. Further tests on the effect of each feature suggested that this confusion is mainly induced by AccZ (it adds some non-informative noise). Thus, we removed it and finally trained the neural network with three descriptors: AccX, AccY and DBA. Finally, we tested the model on three green turtles, never seen by the model before, computing the confusion matrix, the global accuracy, the Recall and Precision indicators relative to each of the behaviors as in Jeantet et al. [24].

### 2.4. Estimation of Laid Eggs

**Figure 2:**
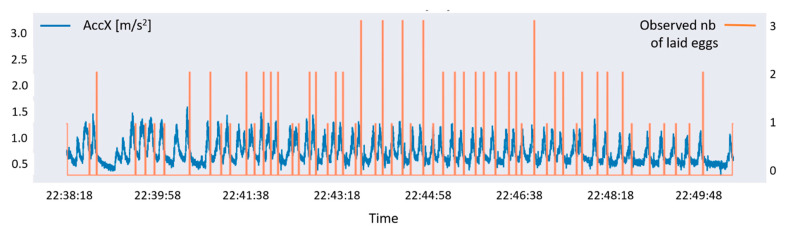
Visualization of the surge acceleration axis (back-to-front or X-axis, in blue) of one green turtle associated with the number of laid eggs counted in the field (in orange).

Once the V-net has predicted the six behavioral categories, it became possible to automatically extract the predicted Egg laying stage and to estimate the number of laid eggs. The laying process is associated with a very slight back and forth movement of the sea turtle’s body which can be visualized on the X-axis of the accelerometer. Its visualization synchronized with the observed number of laid eggs in the field suggested that a peak on the X-axis acceleration signal corresponded to a contraction (Figure 2). Thus, the number of eggs, related to the number of contractions, was estimated by detecting the number of peaks expressed on the X-axis acceleration signal. Some contractions expressed by the green turtles may be associated with the absence of egg deposition, but they were in the minority and occurred mostly at the end of the egg laying process. Due to their low number, we did not consider these contractions. The hypothesis that the number of eggs laid during one contraction depending on the intensity of that contraction, and thus the associated peak, was also considered, though was not conclusive (Figure 2).

#### 2.4.1. Cutting off the Egg Laying Period

**Figure 3:**
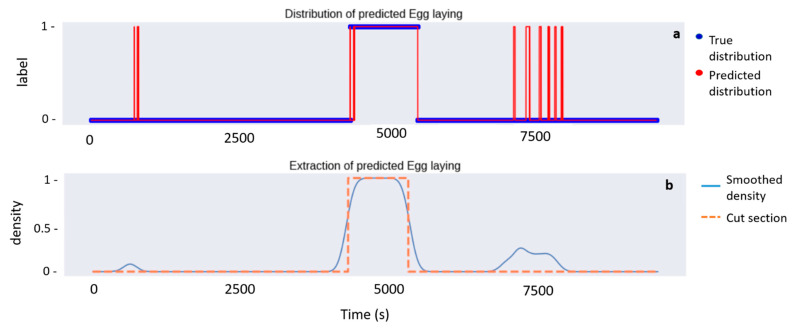
Representation of each step of the extraction of the Egg laying period from the predictions of the V-net for the individual #11. The first panel (**a**) shows the true distribution of Eff Laying over time compared to the predicted distribution by the V-net. The second panel (**b**) shows the smoothed signal of the predicted distribution while the orange dashed line represents the automatically extracted Egg Laying period from which the number of eggs laid is estimated.

To automatically extract the accurate Egg laying part from the V-net predictions, we first discarded the false positive identifications, which generally corresponded to very short sequences distributed in the nesting sequence. For this purpose, we performed the next algorithm with each step depicted in Figure 3:

1. Binarize the behaviors sequence: label “1” is assigned to the behavior Egg laying while all the others are labelled as “0” (Figure 3a);
2. Perform a convolution of the binarized sequence with a Gaussian mask whose standard deviation is empirically chosen. The convolved signal is represented in blue as the ‘Smoothed density’ (Figure 3b);
3. Choose a minimal threshold (threshold = 0.7), and extract the acceleration values associated to the part of the convolved signal which is greater than it (Figure 3b).

#### 2.4.2. Peak Detection

At this point, as it has been concluded that X-axis acceleration contained the largest amount of information for estimating the number of eggs laid, the next analysis was only performed on this axis. In order to augment the precision of peak detection, we firstly smoothed the extracted Egg laying signal using a narrow Gaussian mask. Moreover, we observed a decrease of the average values of the signal all over the laying process, with lower peaks at the end, making their identification difficult compared to the higher peaks at the beginning. We corrected this by subtracting from the trend from its signal, estimated by a second-degree polynomial, adjusted by least-squares approximation. The data are also centered with respect to its average values inside the Egg laying category.

**Figure 4:**
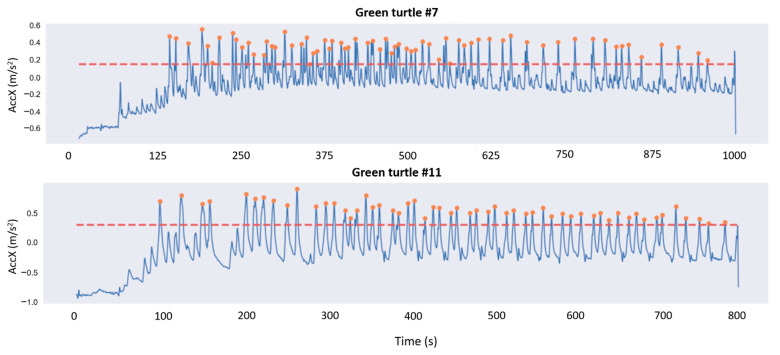
Visualization of the surge acceleration axis (back-to-front or X-axis, in blue) of the laying process of two green turtles with the peaks detected from a rolling window with width of 200.

To estimate the number of peaks over the X-axis, assumed to be related to the number of turtle contractions, we ran over the signal a rolling window with a width approximatively equal to the distance between two picks and detected the local maximum for each window. To avoid detecting the same maximum several times, we kept the value only if it was located in the very middle of the rolling window. Finally, a threshold parameter (represented in dotted red in Figure 4) was chosen as a proportion of the median of the signal. Every found local maximum under this threshold was discarded (Figure 4).

#### 2.4.3. Estimation of the Number of Eggs

We used the estimated number of contractions to calculate the number of laid eggs. From the egg numbers per contraction recorded in the field (from one to four eggs), we calculated the mean number of eggs laid per contraction per turtle and obtained an average of 1.6 (standard deviation = 0.05). For each turtle, we multiplied the estimated number of contractions by this mean to obtain the estimated number of eggs laid. The mean number of eggs laid per contraction should be reconsidered in a larger population to improve its accuracy.

We tested the entire procedure (from the V-net identification to the estimation of number of laid eggs) on the eight green turtles distributed in the training/validation/testing dataset for which the number of laid eggs has been observed.

## 3. Results

**Figure 5:**
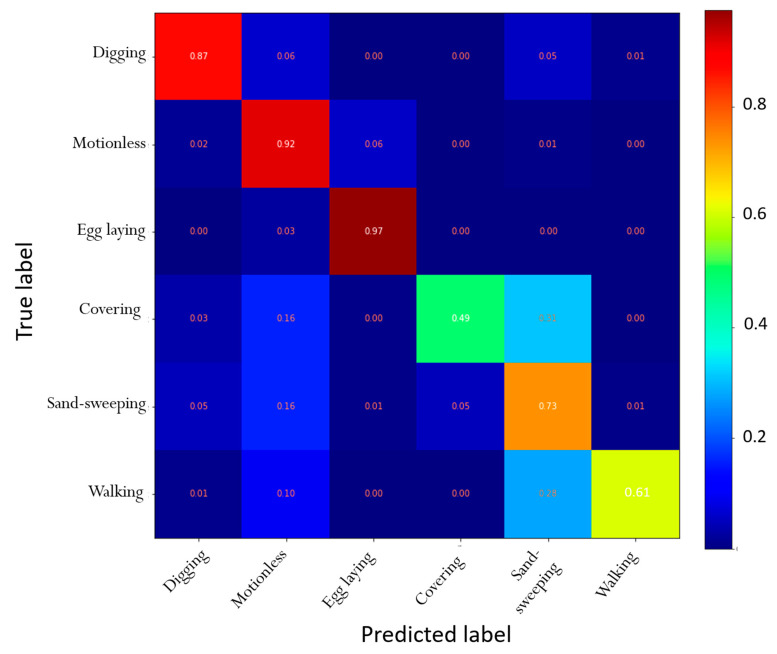
Confusion matrix of the predictions obtained from the V-net for the three green turtles of the validation dataset.

**Figure 6:**
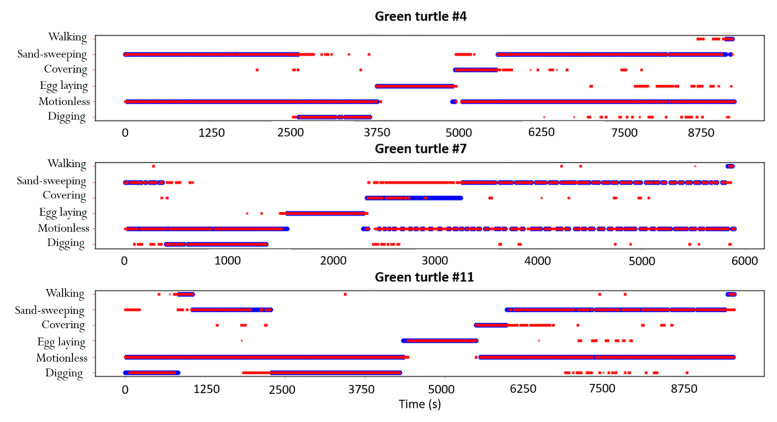
Activity budget of the three green turtles of the validation dataset showing the behaviors inferred by the V-net (in red) compared to actual behaviors (in blue).

The V-net predicted the six behaviors (Sand-sweeping, Digging, Egg laying, Covering, Walking and Motionless) with an accuracy of 95%. It correctly identified 97% of the Egg laying dots, corresponding to the highest Recall index (Figure 5, Table 2). The lower Precision index for this behavior (0.79%) was due to Motionless dots being wrongly predicted as Egg laying. However, since the latter occured one time during the nesting process and was very well identified by the V-net, the Egg Laying period clearly differed from the other behaviors when visualizing the activity budget (Figure 6). The misidentifications from the V-net concerned more Covering and Walking that were confused with Sand-sweeping, leading to the lowest Recall and Precision indexes for these two behaviors (Figure 5, Table 2). The visualisation of the activity budget revealed that it was mostly the end of the Covering process that was confused with Sand-sweeping. (Figure 6).

The correct identification of Egg laying allowed its automatic extraction with sufficient precision to estimate the number of contractions. Thus, from the V-net predictions, we succeeded in estimating the number of eggs with a mean relative error of 7% (standard deviation = 0.06, Table 3).

## 4. Discussion

This study provides the first method to automatically determine the reproductive outputs of the nesting process of green turtles, from animal-attached accelerometers. Using deep learning, we firstly identify the six behaviors expressed by the individuals (Sand-sweeping, Digging, Egg laying, Covering, Walking and Motionless) with an accuracy of 0.95 and a precise detection of the Egg Laying process (Recall index: 0.97). In a second step, we estimate the number of laid eggs from the predicted Egg Laying sequence and find the reproductive outputs with a mean relative error of 7%. The main aim of this method is to support field monitoring of nesting sea turtles by providing a remote method and thus reducing the monitoring effort. In the interests of improving our knowledge of sea turtles, we expect that this method will be a valuable tool for measuring maternal investment in sea turtles and understanding the parameters that influence it.

### 4.1. Automatic Identification of Nesting Behaviors

The V-net is a powerful algorithm that successfully identifies the six behaviors of the nesting process of the green turtles from the accelerometer with an accuracy of 0.95. Similarly, Nishizawa et al. [23] performed the same task using a Classification and Regression Tree (CART) and obtained an accuracy of 0.86 for the same behavioral categories, but without Motionless. Thus, the V-net represents a major improvement as it does not require pre−processing (no segmentation and hand−crafted feature extraction), while having a better accuracy than the CART. Moreover, this study is the second one to use a V-net to perform behavioral identification from the acceleration signals of green turtles (at sea, [24]). The fact that we used the same architecture, and the same hyper−parameters, on similar but not identical data was a positive time saver, which is also promising for future works using loggers.

The main confusion from the V-net concerns Covering and Sand-sweeping. The visualisation of the activity budget shows that this misclassification appears between the end of Covering and the beginning of Sand-sweeping. This confusion is mainly due that nesting turtles express rear flipper sweeping movements in the two stages [28]. In fact, Covering ends with rear flipper sweeps consecutively to rear knead movements, while the following Sand-sweeping stage begins with simultaneous both rear and front flipper sweeps and is characterised by sweeps of the front flippers alone at the end. Nishizawa and al. [23] also obtained the lowest Recall index associated with Covering. Confusions on behavioral identification from supervised learning algorithms were also revealed on other species for which different behaviors encompass similar mechanistic movements [32,33,34]. More generally, the automatic behavioral identification from accelerometer are based on the animals’ posture and the movements and thus requires the precise definition of the behavioral categories based on these, rather than the function or action of the behaviors. In our case, a more precise identification and annotation of the movements involved in Covering and Sand-sweeping in the field (such as ‘rear flipper sweeping’, ‘front flipper sweeping’ and ‘covering’) would probably improve the precision of the V-net for those two behaviors. However, the main challenge in remote monitoring of sea turtles during the breeding season is to detect the egg laying process because in marine turtles, and more markedly in green turtles, individuals come ashore several times in the same night before laying eggs [10,15]. This is why it is important to detect with certainty if the turtle has laid eggs or not and to understand the reasons for these U−turns. Our study allowed us not only to detect the six behavioral categories of the nesting process, but also a more accurate detection of the Egg laying process by the V-net (Recall index = 0.97).

After this step, the second challenge was to automatically estimate the number of eggs laid, which would thus make it possible to determine the maternal investment during one nesting season.

### 4.2. Automatic Identification of Number of Eggs Laid

This study is the first to propose a fully automatic method to remotely estimate the number of laid eggs from a bio-logger. The precise detection of the Egg laying process allowed us to automatically extract the associated acceleration signals and estimate the number of eggs laid. We succeeded in estimating the number of eggs laid with a mean relative error of only 7%. However, it remains difficult to identify the main causes of error considering underestimates of the number of eggs laid for some individuals and overestimates for others (Table 3). The parameters that may lead to over- or underestimation are the accuracy of the associated acceleration sequence extraction, the thresholds fixed to identify the number of peaks and the mean number of eggs laid per contraction obtained from field observation (1.6 ± 0.05). The latter is rather constant with an exact value between 1.57 and 1.59 for the three individuals associated with a relative error above 10%. In all cases, these estimation errors remain low with relative errors below 15% for most individuals and highlight the potential of this method for remote monitoring of sea turtles on land during nesting season.

### 4.3. Perspective of Application

The main aim of the proposed method is, therefore, to support field nesting sea turtles’ monitoring while reducing the monitoring effort, via the remote monitoring of nesting sea turtles for estimation of maternal investment. In particular in French Guyana, given that we know the average number of spawns per individual per season for green turtles and the average delay between two successive nesting processes (Chevallier, personal observations), it would become possible to equip several dozen females with bio-loggers at the start of the breeding season and recover them at the estimated end of their nesting season. Therefore, we would go from exhaustive monitoring 7 days a week during 6 months to 30 days of patrols (5 days to equip and 25 days to recover the materials with a large margin of error on the last return of the green turtles to avoid missing them). Although further research is needed to determine the impact of equipment attached to turtles, the miniaturization of the accelerometer allows for miniature loggers (weight less than 5 g and size 22 × 13 × 8 mm, http://www.technosmart.eu, accessed on 15 February 2022) making this long tracking feasible. Therefore, this long term monitoring of sea turtles from bio-loggers during the whole breeding period would allow researchers to know precisely the clutch frequency, its clutch size and variation during the breeding season for a representative part of a population, and therefore the estimation of their maternal investment, while reducing the patrol time.

Furthermore, the estimation of the reproductive effort of nesting females on land is complementary to the use of the accelerometer on green turtles at sea. Indeed, the proposed method is part of a more general framework where a validation and automatic identification of the underwater behaviors of green turtle from accelerometer data have already been achieved [22,24]. It would then be possible, using accelerometers deployed over the entire breeding season, to describe the underwater behaviors expressed by green turtles, during two successive nesting processes, i.e., the intra−nesting period, and to estimate the number of laid eggs on land. All this information is essential to study the cause−effect relationships between the energy strategy undertaken at sea and the maternal investment. Indeed, inter- and intra-population variations in reproductive outputs have been observed suggesting the influence of the environmental resource availability and the fitness of the individuals [2,4,35]. Whereas the clutch frequency and size are indicative of the success or failure of the individual’s energetic strategy in response to the environmental conditions, the identification of the underwater behaviors enable the identification of this strategy during the inter-nesting period. Combined with environmental data (food availability, water temperature, and ocean current), it could help researchers to identify the extent to which environmental factors influence this energetic strategy and thus the maternal investment. The parallel monitoring at sea and on land could be a key parameter for understanding the adaptive capacities of marine turtles in the context of climate change.

## 5. Conclusions

This experimental study initiates the first steps towards an efficient method of the sea turtles’ reproductive outputs recording from low-cost miniature sensors. Such an approach allows noticeable reduction of monitoring effort and minimizing of human error.

Recovery of bio-loggers, few weeks later, can still be tedious work, but the development of satellite-relay data tags with on-board processing represents a promising alternative. Indeed, it is already possible to remotely transmit a summary of the tri-axial acceleration from satellite-relay data tags [36,37,38] and to implement the learning algorithm into the logger [39]. This next step would enable the researchers to remotely, and almost in real time, follow the nesting behaviors of the equipped individuals (with the estimation of the number of eggs laid) and to relate this information with their behaviors at sea over long periods (pre−nuptial migration, breeding season, post−nuptial migration).

All of these associated technologies will allow the acquisition of acquire knowledge that has never been obtained until now, of the influence of marine environmental parameters on individual’s behavior at sea over long periods (migrations) and the consequences on their maternal investment during reproduction periods. This challenge seems very accessible in the near future.

## Figures and Tables

**Table 1:** Summary of the nesting green turtles’ measures and the observed number of laid eggs. CCL= Curved Carapace Length, CCW= Curved Carapace Width. The dashes indicate the individuals for which the number of laid eggs could not be counted.

Individual	CCL	CCW	First Recorded Behavior	Nb of Laid Eggs	Comments
**#1**	126	122	Egg laying	-	
**#2**	111	103	Digging	-	
**#3**	122	109	Sand-sweeping	-	
**#4**	112	96	Sand-sweeping	-	
**#5**	115	110	Digging	106	
**#6**	114	113	Digging	111	
**#7**	102	94	Digging	93	
**#8**	112	94	Sand-sweeping	117	
**#9**	108	98	Digging	103	
**#10**	128	110	Digging	173	
**#11**	119	104	Sand-sweeping	93	
**#12**	105	96	Sand-sweeping	-	Did not lay eggs
**#13**	117	104	Digging	-	
**#14**	118	106	Sand-sweeping	97	

**Table 2:** Recall and Precision index obtained for the six nesting behaviors from the V-net for the three green turtles of the validation dataset. Accuracy (in bold) measures the ability of the V-net to correctly identify all behaviors as a whole.

	Recall	Precision
Digging	0.87	0.79
Motionless	0.92	0.90
Egg laying	0.97	0.79
Filling and packing	0.49	0.72
Sand-sweeping	0.73	0.84
Walking	0.61	0.70
**Accuracy**		**0.95**

**Table 3:** Estimations of the number of laid eggs for eight green turtles from the Egg laying period identified by the V-net and/or manually extracted from the acceleration visualization compared to the actual observed numbers on the field.

Individual	Nb of Observed Eggs	Nb of Estimated Eggs	Difference	Relative Error
#5	106	101	−5	0.05
#6	111	109	−2	0.02
#7	93	93	0	0.00
#8	117	118	1	0.01
#9	103	117	14	0.14
#10	173	150	−23	0.13
#11	93	88	−5	0.05
#14	97	112	15	0.15
MEAN			−1	0.07

## Data Availability

Not applicable
